# Supplementary material for: Exploration of the Biocontrol Activity of Bacillus atrophaeus Strain HF1 against Pear Valsa Canker Caused by Valsa pyri
Source: Int J Mol Sci. 2023 Oct 23;24(20):15477. doi: 10.3390/ijms242015477 (PMC10607598; doi:10.3390/ijms242015477)
Supplement: Supplementary file 1 [file ijms-24-15477-s001.zip › Figures.pdf]

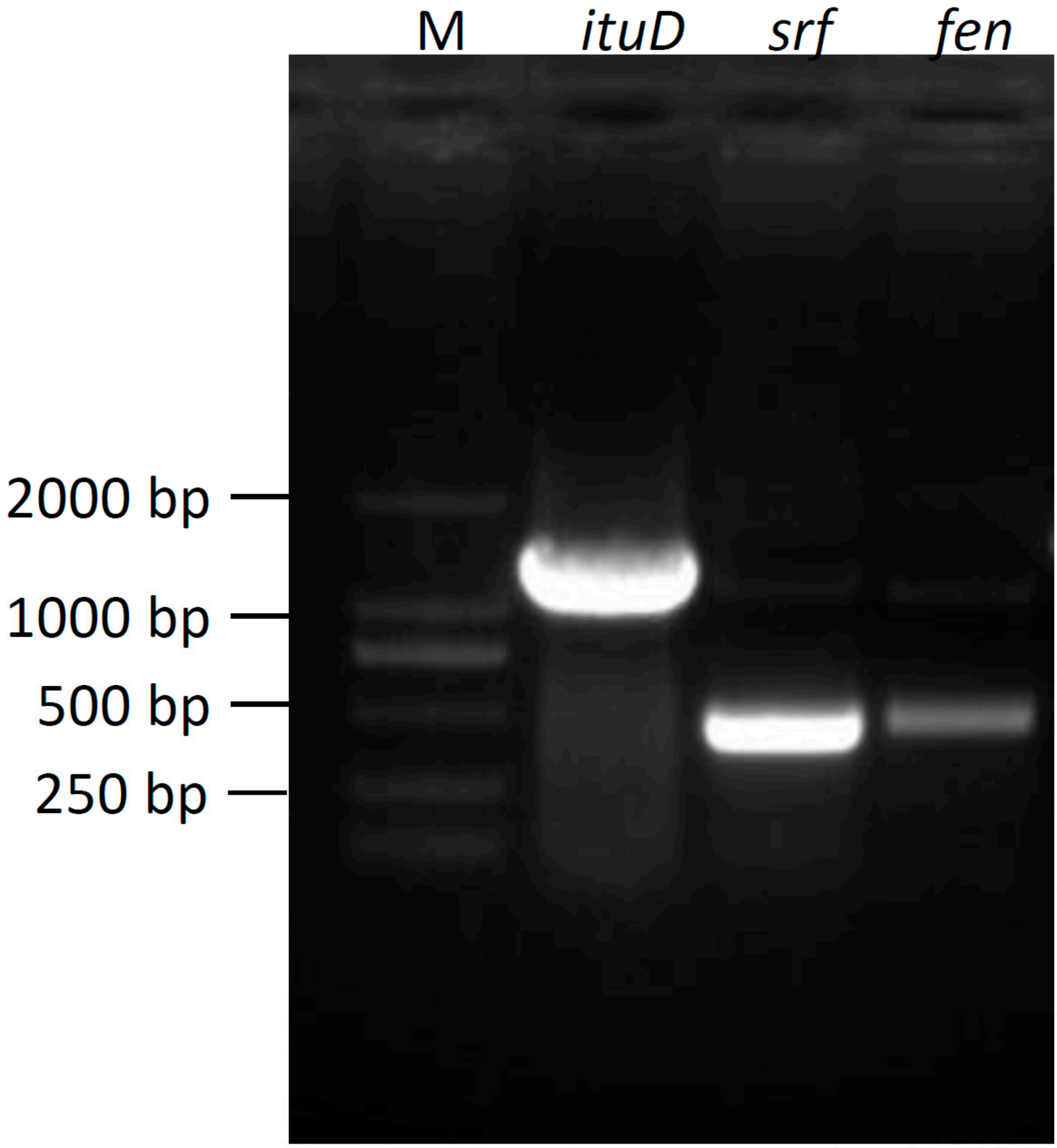


Figure S1: Amplification of antibiotic biosynthesis genes in *B. atrophaeus* strain HF1.

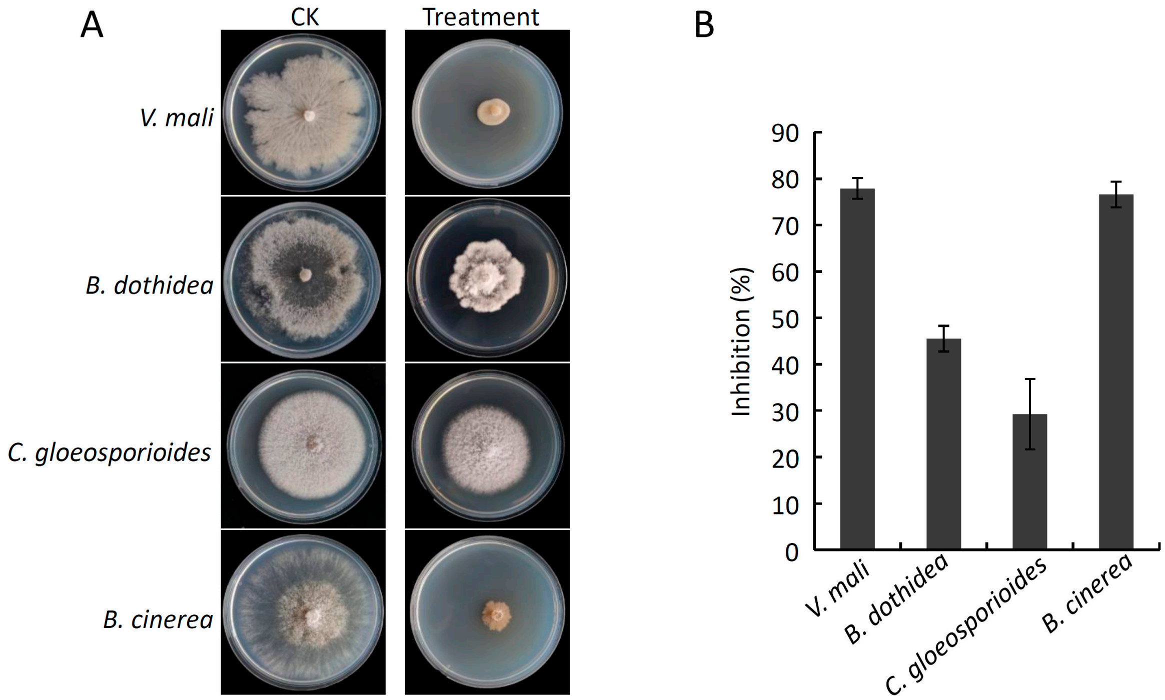


Figure S2: Effect of *B. atrophaeus* strain HF1-produced VOCs on other fruit pathogens growth. (**A**) *V. pyri* colony morphology after fumigation with 100 μL of 1 × $10^8$ CFU $mL^{-1}$ of strain HF1 cell suspension. (**B**) Inhibition rate. Each dataset is expressed as the mean ± SD of three biological replicates. Different lowercase letters denote significant differences at $P < 0.05$ between different treatments. The experiment was performed independently three times with similar results.
