# Supplementary material for: Exploration of the Biocontrol Activity of Bacillus atrophaeus Strain HF1 against Pear Valsa Canker Caused by Valsa pyri
Source: Int J Mol Sci. 2023 Oct 23;24(20):15477. doi: 10.3390/ijms242015477 (PMC10607598; doi:10.3390/ijms242015477)
Supplement: Supplementary file 1 [file ijms-24-15477-s001.zip › Tables.pdf]

Table S1. Physiological and biochemical characters of strain HF1

Test	Reaction[a]
Gram stain	+
Voges-Proskauer (VP) test	+
Gelatin liquefaction	+
Methyl red test	−
Nitrate reductase	+
Starch hydrolysis	+
Protease	+
Chitinase	+
Sucrose fermentation	+
Xylose fermentation	−
Mannitol fermentation	−

[a] + and − represent positive and negative reactions, respectively. Data within each column are the means of three independent experiments.

Table S2. Toxicity test of three pure compounds on *V. pyri*

Pure compounds	EC_{50} (μL L^{-1})	Regression equation	Correlation coefficient
Hexanoic acid	6.65	y=2.9129+2.5357x	0.9872
Iberverin	0.30	y=7.2351+4.2403x	0.9416
2-Methylvaleraldehyde	74.07	y=-18.4226+12.5278x	0.9642

Table S3. Primers used in this study

Primer	Primer sequence (5′-3′)	Gene
SR1	AAGGAGGTGATCCAGCCGCA	16S rDNA
SR2	AGAGTTTGATCCTGGCTCAG	
gyrA_F	CAGTCAGGAAATGCGTACGTCCTT	*gyrA*
gyrA_R	CAAGGTAATGCTCCAGGCATTGCT	
groEL-550f	GAGCTTGAAGTKGTTGAAGG	*groE*
groEL-1497r	TGAGCGTGTWACTTTTGTWG	
ituD-F	ATGAACAATCTTGCCTTTTTA	*ituD*
ituD-R	TTATTTTAAAATCCGCAATT	
Sur-F	CGCGGMTACCGVATYGAGC	*srf*
Sur-R	ATBCCTTTBTWDGAATGTCCGCC	
Fen-F	GAATAYMTCGGMCGTMTKGA	*fen*
Fen-R	GCTTTWADKGAATSBCCGCC	
